# Predicting Postoperative Survival in Patients With Malignant Biliary Obstruction Using an Interpretable Machine Learning Model: A Multicenter Study

**DOI:** 10.1002/cam4.71692

**Published:** 2026-03-06

**Authors:** Zongdong Zhu, Chenyang Bian, Linjing Zhao, Weiwei Li, Kaixin Hu, Qinggao Song, Huangbao Li

**Affiliations:** ^1^ Zunyi Medical University Zunyi Guizhou China; ^2^ Jiaxing University Master Degree Cultivation Base Zhejiang Chinese Medical University Jiaxing Zhejiang China; ^3^ Department of Hepatobiliary and Pancreatic Surgery, The First Hospital of Jiaxing, Affiliated Hospital of Jiaxing University Jiaxing Zhejiang China; ^4^ Affiliated Hospital of Shaanxi University of Traditional Chinese Medicine Shaanxi University of Traditional Chinese Medicine Xianyang Shaanxi China; ^5^ The Second Hospital of Jiaxing Jiaxing Zhejiang China; ^6^ Hospital of Stomatology Zunyi Medical University Zunyi Guizhou China

**Keywords:** biliary drainage, endoscopic retrograde cholangiopancreatography, machine learning, malignant bile duct obstruction

## Abstract

**Background:**

Endoscopic bile duct drainage is crucial for improving the survival and quality of life in patients with malignant biliary obstruction (MBO). This study aimed to identify key factors that affect postoperative survival in patients with MBO, develop a predictive model, and validate the performance of the model using external data.

**Methods:**

Data were retrospectively collected from The First Hospital of Jiaxing (between 2013 and 2021), The Second Hospital of Jiaxing (between 2014 and 2021), and Affiliated Hospital of Shaanxi University of Traditional Chinese Medicine (between 2014 and 2021). Patient demographics, disease characteristics, and laboratory results of 337 patients were analyzed. Various machine learning models, including the gradient boosted survival tree, extreme gradient boosting (XGBoost), XGBoost accelerated failure time (XGBoost AFT), random survival forests, and Cox proportional hazards regression, were used. The model performance was assessed using the concordance index (C‐index), and SHapley Additive exPlanations (SHAP) values were used to interpret the model.

**Results:**

The XGBoost AFT model exhibited the best performance, with C‐index values of 0.902, 0.722, and 0.705 for the training cohort, test cohort 1, and test cohort 2, respectively. The SHAP analysis revealed that distant metastasis, high total bilirubin level, prolonged prothrombin time, and high‐level obstruction significantly impacted survival. A Kaplan–Meier survival analysis demonstrated that the model effectively stratified patients into the high‐risk and low‐risk groups.

**Conclusions:**

This study provides a robust model that can predict the postoperative survival in patients with MBO. This model was validated using external data, and the results offer valuable guidance for postoperative management and personalized treatment.

**Abbreviations**
C‐index: concordance index
CI: confidence interval
GBST: gradient boosted survival trees
HR: hazard ratio
MBO: malignant biliary obstruction
ML: machine learning
RSF: Random Survival Forests
SHAP: Shapley additive explanations
VIF: variance inflation factor
XGBoost AFT: extreme gradient boosting accelerated failure time
XGBoost: extreme gradient boosting

## 1 Background

Malignant biliary obstruction (MBO) is a condition caused by malignant tumors that leads to bile duct obstruction [1, 2, 3, 4]. Jaundice, which results from bile duct blockage, is the primary initial symptom of MBO [5]. Because of the lack of specific symptoms during the early stages of disease, most patients are diagnosed at an advanced stage when curative surgery is not feasible [6, 7, 8]. Endoscopic biliary drainage is the first‐line treatment for patients with MBO [2, 9, 10]. Early and effective palliative biliary drainage for patients with malignant obstructive jaundice can significantly improve their survival rates and quality of life [10].

Although biliary drainage is necessary for patients with MBO, their prognosis remains unsatisfactory [11]. The use of large‐scale patient data to explore factors that influence the prognosis of MBO and establish predictive models can potentially guide clinical decisions and improve outcomes. Previous studies have primarily focused on univariate and multivariate analyses to assess the prognosis based on a limited number of clinical indicators [12, 13, 14, 15]. Furthermore, most MBO prediction models have been developed using traditional statistical methods such as Cox proportional hazards regression or logistic regression [7, 16, 17].

With advances in computer science, machine learning (ML) is being increasingly applied in the medical field. ML excels at handling large, complex, and heterogeneous data, and many studies have demonstrated that ML models exhibit predictive performance that is superior to that of traditional models [18, 19, 20, 21]. However, in the field of ML, particularly ML with complex models such as deep neural networks and ensemble models (e.g., random forests and gradient boosting trees), the decision‐making process of these models is often difficult to interpret [22]. This “black box” nature limits their applications in many critical areas. To address this issue, the SHapley Additive exPlanations (SHAP) method has been recently introduced [23, 24]. The SHAP method allows for the identification and prioritization of attributes influencing complex classifications and predictions using any ML model. Therefore, developing a visualized predictive model that can help healthcare professionals identify individuals with a poor prognosis would be highly beneficial [25].

In this study, we explored factors influencing the prognosis of patients with MBO, established a reliable ML model, and validated the generalizability of that model using external data. We also used an interpretable algorithm for the ML model to interpret its predictions.

## 2 Methods

### 2.1 Patient Selection

This study retrospectively collected patient data from The First Hospital of Jiaxing (institution 1; between June 1, 2013, and August 31, 2021); The Second Hospital of Jiaxing (institution 2; between June 1, 2014, and September 30, 2021); and Affiliated Hospital of Shaanxi University of Traditional Chinese Medicine (institution 3; between June 1, 2014, and September 30, 2021). A total of 626 individuals participated in this study.

### 2.2 Inclusion Criteria

Patients were included in the study if they met the following criteria:

1. Diagnosed with malignant tumors caused by tumor invasion and confirmed by pathological or imaging examinations;
2. Diagnosed with primary or secondary liver, bile duct, gallbladder cancer, pancreatic cancer, or periampullary malignant tumors;
3. Older than 18 years of age; and
4. Planed to undergo endoscopic retrograde cholangiopancreatography (ERCP) for the first time and intend to have a biliary metal stent implanted.

### 2.3 Exclusion Criteria

Patients were excluded if they met any of the following criteria:

1. Disease was diagnosed at a resectable stage;
2. Have a history of common bile duct surgery;
3. Follow‐up duration of less than 30 days because of hospital transfer or other reasons; and
4. Failed cannulation or no stent placement.

Patients with a history of common bile duct surgery were excluded because the altered anatomical structure may affect the technical success rate of ERCP, the choice of stents, and the postoperative survival outcomes, potentially having a confounding effect on the performance of the predictive model.

**FIGURE 1:**
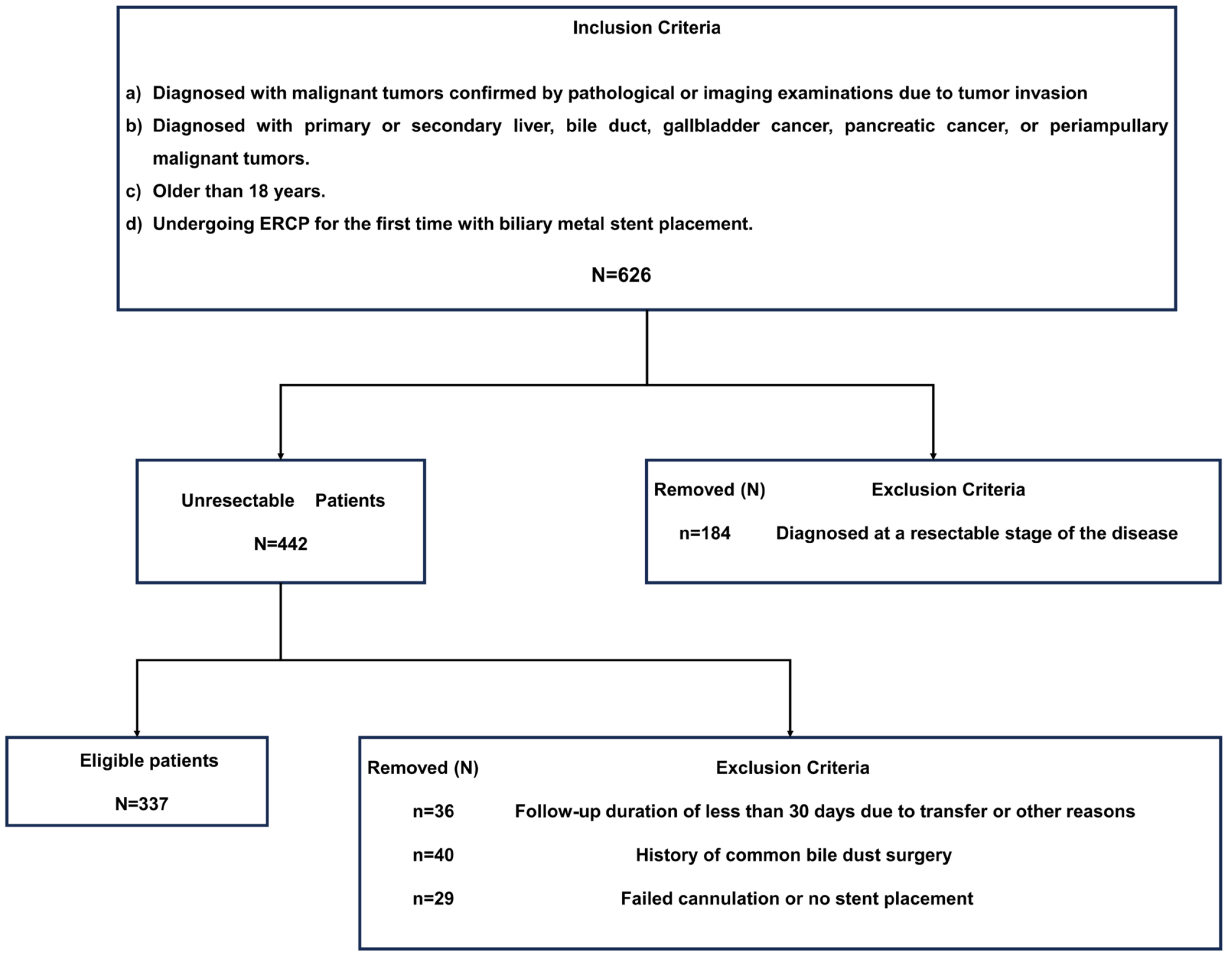
Detailed outline of the patient screening process based on the inclusion and exclusion criteria. ERCP, endoscopic retrograde cholangiopancreatography.

Based on these criteria, 337 participants were included in this study (Figure 1). The study protocol adhered to the ethical guidelines of the 1995 Declaration of Helsinki and was approved by the ethics committees of The First Hospital of Jiaxing (ethics approval no. 2022‐LY‐229), The Second Hospital of Jiaxing (ethics approval no. 2023‐ZFYJ‐066), and Affiliated Hospital of Shaanxi University of Traditional Chinese Medicine (ethics approval no. SZFYIEC‐YJ‐2024‐146). The requirement for informed consent was waived for the retrospective cohort.

### 2.4 Data Collection

A retrospective analysis of various parameters such as basic demographic information (age, sex), disease characteristics (tumor type, ascites, obstruction site, distant metastasis, lymph node metastasis, cholangitis before stent insertion, Child–Pugh class), laboratory test results (white blood cell count, red blood cell count, hemoglobin level, platelet count, total bilirubin level, alanine aminotransferase level, aspartate aminotransferase level, albumin level, prothrombin time, creatinine level), and the number of stents was conducted. Due to differences in standards and completeness among various centers in recording immunonutritional indicators such as lymphocyte count and prognostic nutritional index, in order to ensure data consistency, the above‐mentioned variables were not included in this study. Follow‐up procedures included all outpatient evaluations, hospital appointments, and telephone consultations performed until December 31, 2022.

### 2.5 Definitions

High obstruction refers to obstruction between the common hepatic duct and intrahepatic bile ducts, whereas low obstruction refers to obstruction within the common bile duct or ampulla. Distant metastasis was defined as M1 and lymph node metastasis was defined as N1 or higher, according to the American Joint Committee on Cancer 8th edition TNM staging system. Cholangitis before ERCP is defined as the pre‐existing biliary tract infection before the procedure. All laboratory parameters were obtained from the most recent test results prior to ERCP. The survival time of patients was calculated from the date of ERCP until death or the end of the follow‐up period (in days).

### 2.6 Outcomes

The primary outcome measure of this study was the overall survival time of patients after stent placement (i.e., after ERCP).

### 2.7 Statistical Methods and Model Development

Given the retrospective design of the study, no a priori power analysis was conducted. However, based on the rule of thumb in predictive model development that “at least 10 events per variable” was used to perform a post‐hoc assessment of the sufficiency of the sample size, it was confirmed that the sample size met the requirements for model construction.

Statistical analyses and model development were performed using R version 4.3.0. To address missing data, multiple imputation was performed using the mice package (version 3.16.0) in R, generating five imputed datasets. The imputation model incorporated all covariates and outcome variables relevant to model development, including preoperative infection, Child‐Pugh grade, white blood cell count, red blood cell count, hemoglobin, platelet count, total bilirubin (TBIL), alanine aminotransferase (ALT), aspartate aminotransferase (AST), albumin, creatinine, prothrombin time (PT), as well as survival time and status. For continuous variables, the predictive mean matching (PMM) method was employed. The missing rate of all variables was less than 5%, which is at a low level. The multiple imputation method can provide a reliable estimate of uncertainty while retaining the sample size [26, 27]. The distributions of all continuous variables, particularly total bilirubin and prothrombin time, were rigorously screened for potential outliers. Although certain values were observed at the distribution tails, they were retained to preserve biological variability and ensure the model's clinical utility in critically ill patients, given their physiological plausibility within the context of malignant biliary obstruction (MBO) and the absence of data entry errors. To evaluate the robustness of our findings, a sensitivity analysis was conducted: outliers (defined as values exceeding Q1–3\times IQR or Q3 + 3\times IQR) were replaced with median values, and the model was refitted. The results confirmed that the model's discriminative performance remained robust (Figure S1). All predictive variables were standardized for the analysis. Categorical variables are presented as percentages and frequencies, whereas continuous variables are expressed as mean ± standard deviation for normally distributed data and as interquartile range for skewed data. The chi‐square test or Fisher's exact test was used to compare categorical variables, whereas the Mann–Whitney U test was used for continuous variables. A *p* value of < 0.05 was considered statistically significant.

A univariable Cox regression analysis was performed to identify clinical variables influencing the study outcomes; variables were compared between patients who survived and those who did not. Hazard ratios and 95% confidence intervals (CIs) were calculated for all variables. Significant variables from the univariable analysis were included in the multivariable Cox regression model. Variables deemed significant in the multivariable Cox regression analysis were considered independent risk factors affecting the outcome.

Feature selection was executed using the least absolute shrinkage and selection operator (LASSO) regression to mitigate potential overfitting or underfitting. Multicollinearity among the selected variables was assessed using the variance inflation factor (VIF), with a VIF > 5.0 indicating significant multicollinearity. Variables exhibiting high VIF values were excluded from the final analysis. The ultimate predictive model was synthesized based on LASSO regression results, multivariable Cox regression analysis, clinical expertise, and VIF assessments. To further optimize performance and control overfitting, for each machine–learning algorithm (Gradient Boosted Survival Tree, Extreme Gradient Boosting, XGBoost Accelerated Failure Time, Random Survival Forest), grid search combined with 5–fold cross–validation within the training cohort was used for hyperparameter tuning. Taking XGBoost AFT as an example, the main parameters adjusted were the learning rate (0.01–0.3), maximum tree depth (3–10), row sampling ratio (0.6–1.0), and column sampling ratio (0.6–1.0). For other tree–based models, the focus was on optimizing tree structure parameters, the number of iterations, and regularization coefficients. During the training process, an early–stopping strategy was set (termination when the performance of the validation set did not improve for 50 consecutive rounds), and L1 and L2 regularization (with coefficient ranges from 0 to 1.0) were introduced to constrain the model complexity. The detailed hyperparameter settings for each algorithm are shown in Table S1.

For a robust benchmarking, we developed a conventional multivariable Cox proportional hazards (CoxPH) model utilizing the same candidate predictors as the XGBoost AFT model to serve as a reference standard. The following five different ML methods were used to select the optimal predictive model: gradient boosted survival tree (GBST), extreme gradient boosting (XGBoost), extreme gradient boosting accelerated failure time (XGBoost AFT), random survival forests (RSF), and Cox proportional hazards (CoxPH) regression. Patients from institution 1 were included in the training cohort, whereas those from institutions 2 and 3 were included in the testing cohort.

### 2.8 Model Performance Evaluation and Model Interpretation

The performance of each model was evaluated using the concordance index (C‐index). The C‐index measures the ability of a model to correctly rank the survival times of patients, with higher values indicating better discriminatory power [28]. We also plotted time‐dependent Receiver Operating Characteristic (ROC) curves to evaluate the discriminative ability and stability of the model in both the training and testing cohorts. The Area Under the Curve (AUC) values range from 0.5 to 1.0, where 0.5 indicates predictions no better than random chance and 1.0 represents a perfect fit. Calibration curves were utilized to assess the model's calibration performance. These curves are valuable tools for validating and optimizing the performance of prognostic models. They typically feature two axes: the *x*‐axis represents the model‐predicted survival probability, while the *y*‐axis represents the actually observed survival probability. If the model's predictions align perfectly with the observed outcomes, the calibration curve should approximate the 45‐degree diagonal line. A substantial deviation from this diagonal indicates bias in the model. The Brier Score (BS) was employed to quantify the squared error between the predicted probabilities and the actual outcomes, with values ranging from 0 to 1, where a lower score indicates higher predictive accuracy. The Integrated Brier Score (IBS), obtained by integrating the BS across the entire observed time period, provides a comprehensive assessment of the model's overall calibration accuracy throughout the study duration.

To interpret the predictive models, we used the SHAP method. The SHAP method provides a unified approach for measuring feature importance in ML models, thus enhancing the visualization and understanding of model predictions [29, 30]. SHAP values are founded on robust theoretical principles and meet the criteria of local accuracy, missingness, and consistency [31, 32]. We used the SHAP method to interpret our predictive model and focused on identifying risk factors associated with the mortality of patients with MBO. This approach aided in understanding the model's predictions and factors affecting patient prognosis.

## 3 Results

### 3.1 Baseline Characteristics

A total of 337 patients were included in this analysis. They were divided into a training cohort and a test cohort according to the research centers. The 245 patients from the First Hospital of Jiaxing (Institution 1) from June 1, 2013 to August 31, 2021 served as the training set. The 40 patients from the Second Hospital of Jiaxing (Institution 2) and 52 patients from Affiliated Hospital of Shaanxi University of Traditional Chinese Medicine (Institution 3), both from June 1, 2014 to September 30, 2021, together constituted the test set (92 patients in total). The average overall survival time post‐surgery was 199 days. Among the patients, 147 (43.6%) were women. Pancreatic cancer was the most common tumor type (158 patients; 46.9%), followed by bile duct cancer (90 patients; 26.7%). High obstruction was noted in 78 (23.1%) patients, and approximately one‐third of the patients had ascites (100 patients; 29.7%). Additionally, approximately one‐tenth of the patients had grade C liver function (33 patients; 9.8%). The baseline characteristics of the three groups are presented in Table 1.

**TABLE 1:** Baseline characteristics of the study groups.

Variables	Training cohort	Testing cohort 1	Testing cohort 2	Total
	(*N* = 245)	(*N* = 52)	(*N* = 40)	(*N* = 337)
Sex, *n* (%)				
Male	142 (58.0%)	27 (51.9%)	21 (52.5%)	190 (56.4%)
Female	103 (42.0%)	25 (48.1%)	19 (47.5%)	147 (43.6%)
Age				
Mean (SD)	72.2 (10.5)	69.0 (11.7)	69.6 (11.3)	71.4 (10.8)
Median [Min, Max]	74.0 [34.0, 91.0]	71.0 [39.0, 91.0]	71.0 [45.0, 90.0]	73.0 [34.0, 91.0]
Obstruction site, *n* (%)				
High obstruction	47 (19.2%)	20 (38.5%)	11 (27.5%)	78 (23.1%)
Low obstruction	198 (80.8%)	32 (61.5%)	29 (72.5%)	259 (76.9%)
Stent placement, *n* (%)				
One stent placement	233 (95.1%)	51 (98.1%)	37 (92.5%)	321 (95.3%)
Two‐stent placements	12 (4.9%)	1 (1.9%)	3 (7.5%)	16 (4.7%)
Ascites, *n* (%)				
No	168 (68.6%)	41 (78.8%)	28 (70.0%)	237 (70.3%)
Yes	77 (31.4%)	11 (21.2%)	12 (30.0%)	100 (29.7%)
Lymph node metastasis, *n* (%)				
No	135 (55.1%)	24 (46.2%)	24 (60.0%)	183 (54.3%)
Yes	110 (44.9%)	28 (53.8%)	16 (40.0%)	154 (45.7%)
Distant metastasis, *n* (%)				
No	158 (64.5%)	29 (55.8%)	25 (62.5%)	212 (62.9%)
Yes	87 (35.5%)	23 (44.2%)	15 (37.5%)	125 (37.1%)
Cholangitis before stent insertion, *n* (%)				
No	200 (81.6%)	48 (92.3%)	33 (82.5%)	281 (83.4%)
Yes	45 (18.4%)	4 (7.7%)	7 (17.5%)	56 (16.6%)
Child‐Pugh class, *n* (%)				
A or B	223 (91.0%)	42 (80.8%)	39 (97.5%)	304 (90.2%)
C	22 (9.0%)	10 (19.2%)	1 (2.5%)	33 (9.8%)
Type of malignancy, *n* (%)				
Cholangiocarcinoma	63 (25.7%)	15 (28.8%)	12 (30.0%)	90 (26.7%)
Gallbladder cancer	24 (9.8%)	11 (21.2%)	10 (25.0%)	45 (13.4%)
Ampullary cancer	24 (9.8%)	1 (1.9%)	1 (2.5%)	26 (7.7%)
Other	15 (6.1%)	1 (1.9%)	2 (5.0%)	18 (5.3%)
Pancreatic cancer	119 (48.6%)	24 (46.2%)	15 (37.5%)	158 (46.9%)
White blood cell count (109/L)				
Mean (SD)	6.67 (4.04)	7.00 (2.89)	8.00 (4.86)	6.88 (4.01)
Median [Min, Max]	6.00 [2.03, 42.9]	6.23 [3.03, 14.3]	6.37 [3.84, 32.6]	6.06 [2.03, 42.9]
Red blood cell count (1012/L)				
Mean (SD)	3.58 (0.605)	3.59 (0.572)	3.81 (0.585)	3.61 (0.601)
Median [Min, Max]	3.61 [1.78, 5.28]	3.60 [1.79, 4.76]	3.89 [2.51, 5.24]	3.62 [1.78, 5.28]
Hemoglobin (g/L)				
Mean (SD)	110 (17.7)	113 (17.4)	116 (20.2)	112 (18.0)
Median [Min, Max]	110 [61.0, 160]	116 [70.0, 160]	118 [70.0, 157]	112 [61.0, 160]
Platelet (109/L)				
Mean (SD)	194 (79.7)	206 (77.5)	228 (93.0)	200 (81.6)
Median [Min, Max]	187 [35.0, 483]	191 [41.0, 418]	217 [85.0, 500]	188 [35.0, 500]
TB (mg/dL)				
Mean (SD)	208 (123)	217 (140)	159 (101)	204 (124)
Median [Min, Max]	201 [5.00, 617]	198 [11.8, 583]	133 [19.1, 437]	179 [5.00, 617]
ALT (u/L)				
Mean (SD)	111 (94.2)	141 (109)	165 (121)	122 (102)
Median [Min, Max]	85.0 [9.00, 848]	103 [16.0, 458]	144 [19.0, 541]	92.0 [9.00, 848]
AST (u/L)				
Mean (SD)	108 (81.0)	139 (97.6)	137 (99.8)	116 (86.8)
Median [Min, Max]	89.0 [16.0, 612]	108 [26.0, 625]	107 [29.0, 410]	94.0 [16.0, 625]
Albumin (g/L)				
Mean (SD)	34.6 (5.24)	33.0 (8.14)	36.1 (6.26)	34.5 (5.93)
Median [Min, Max]	34.7 [22.5, 47.5]	32.2 [19.6, 68.2]	36.6 [22.6, 51.5]	34.7 [19.6, 68.2]
Creatinine (μmoI/L)				
Mean (SD)	69.7 (23.3)	67.9 (13.8)	59.9 (18.4)	68.2 (21.7)
Median [Min, Max]	64.8 [23.6, 203]	67.5 [38.6, 105]	55.5 [35.0, 124]	64.4 [23.6, 203]
PT (s)				
Mean (SD)	14.1 (2.18)	13.6 (1.59)	11.5 (2.03)	13.8 (2.24)
Median [Min, Max]	13.8 [10.9, 35.5]	13.8 [10.0, 19.9]	11.9 [1.21, 14.4]	13.7 [1.21, 35.5]
OS (day)				
Mean (SD)	205 (222)	167 (161)	200 (219)	198 (214)
Median [Min, Max]	140 [2.00, 1840]	117 [17.0, 696]	147 [7.00, 1330]	140 [2.00, 1840]

Abbreviations: ALT, alanine aminotransferase; AST, aspartate aminotransferase; ERCP, endoscopic retrograde cholangiopancreatography; PT, prothrombin time; TB, total bilirubin.

### 3.2 Feature Selection and Model Performance Comparisons

The multivariate Cox regression analysis revealed significant variations in the risk of mortality associated with tumor site, distant metastasis, total bilirubin level, tumor type, and prothrombin time. Specifically, distant metastasis (hazard ratio [HR], 2.47; CI, 1.86–3.27; *p* < 0.01), high total bilirubin level (HR, 1; CI, 1–1; *p* < 0.01), and prolonged prothrombin time (HR, 1.07; CI, 1.01–1.13; *p* = 0.03) were independent risk factors that affected patient survival. Conversely, low obstruction (HR, 0.64; CI, 0.457–0.887; *p* < 0.01) and ampullary cancer (HR, 0.62; CI, 0.399–0.968; *p* = 0.04) were independent factors that protected patient survival (Table 2).

**TABLE 2:** Univariate and multivariate analyses of baseline variables for predicting mortality in the training cohort.

Variants	Univariate analyses		Multivariate analyses	
	HR (95% CI)	*p*	HR (95% CI)	*p*
Female	0.93 (0.72–1.2)	0.58		
Age	1 (0.994–1.02)	0.35		
Low obstruction	0.66 (0.476–0.902)	< 0.01^*^	0.64 (0.457–0.887)	< 0.01^*^
Two‐stent placements	1.5 (0.823–2.64)	0.19		
Lymph node metastasis	1.2 (0.937–1.56)	0.14		
Distant metastasis	2.2 (1.67–2.88)	< 0.01^*^	2.47 (1.86–3.27)	< 0.01^*^
Cholangitis before stent insertion	0.79 (0.563–1.1)	0.16		
Child–Pugh class C	1.9 (1.24–2.99)	< 0.01^*^	1.38 (0.793–2.4)	0.25
Cholangiocarcinoma	0.89 (0.667–1.2)	0.46		
Gallbladder cancer	1.2 (0.784–1.83)	0.40		
Ampullary cancer	0.58 (0.377–0.881)	0.01^*^	0.62 (0.399–0.968)	0.04^*^
Pancreatic Cancer	1.2 (0.966–1.61)	0.09		
Other	1.3 (0.762–2.19)	0.34		
Ascites	1.4 (1.06–1.83)	0.02^*^	1.19 (0.875–1.61)	0.27
White blood cell count (10^9^/L)	1.1 (1.02–1.09)	< 0.01^*^	1.02 (0.986–1.06)	0.23
Red blood cell count (10^12^/L)	0.96 (0.781–1.18)	0.70		
Hemoglobin (g/L)	1 (0.991–1.01)	0.65		
Platelet (10^9^/L)	1 (0.999–1)	0.40		
TB (mg/dl)	1 (1–1)	< 0.01^*^	1 (1–1)	< 0.01^*^
ALT (u/L)	1 (0.998–1)	0.49		
AST (u/L)	1 (0.999–1)	0.99		
Albumin (g/L)	0.97 (0.95–0.998)	0.03^*^	0.99 (0.958–1.01)	0.28
Creatinine (μmoI/L)	1 (0.996–1.01)	0.6		
PT (s)	1.1 (1.02–1.14)	< 0.01^*^	1.07 (1.01–1.13)	0.03^*^
Age	0.93 (0.72–1.2)	0.58		

Abbreviations: ALT, alanine aminotransferase; AST, aspartate aminotransferase; CI, confidence interval; ERCP, endoscopic retrograde cholangiopancreatography; HR, hazard ratio; PT, prothrombin time; TB, total bilirubin.

* Statistical significance at *p* < 0.05.

**FIGURE 2:**
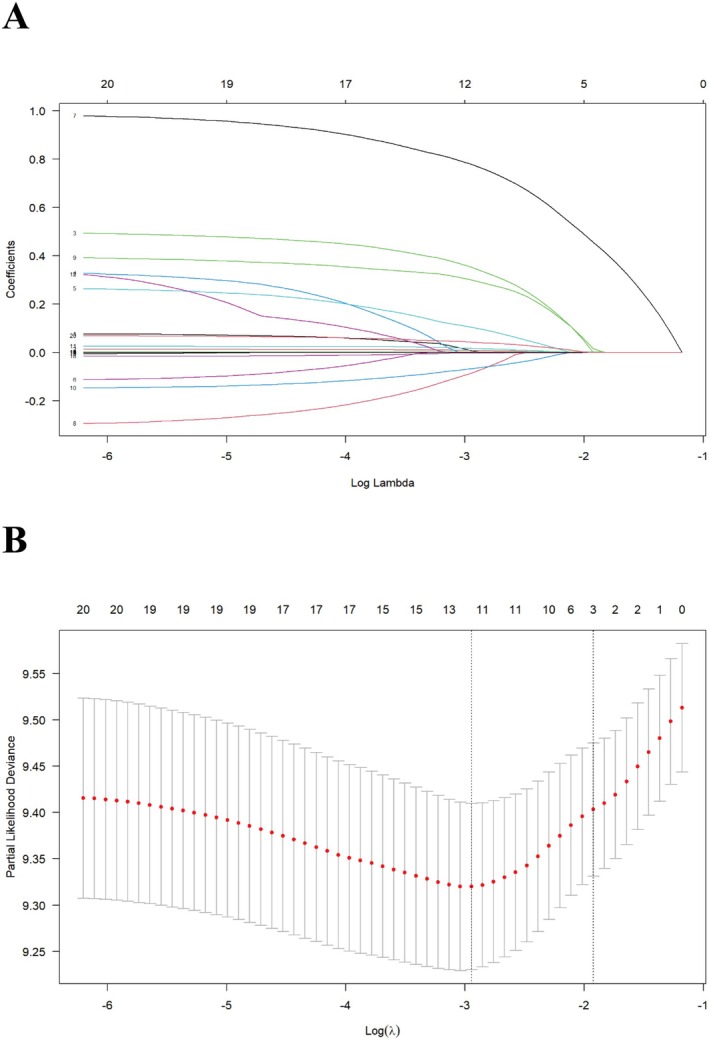
Screening of variables based on the least absolute shrinkage and selection operator (LASSO) regression. (A) Variations of characteristics of the coefficient of variables. (B) Selection of the optimum value of the parameter λ in the LASSO regression model using cross‐validation.

LASSO regression was used to select the parameters, and the coefficient variations of these variables are shown in Figure 2. An iterative analysis with five‐fold cross‐validation identified a high‐performance model with the fewest variables and an optimal λ value of 0.1461465 (Figure 2). Based on these findings, tumor site, distant metastasis, total bilirubin level, and prothrombin time were chosen as predictive factors, accounting for interactions between covariates while highlighting those with a significant statistical impact in the Cox model. All feature VIF values were < 5, indicating no severe multicollinearity among the predictor variables.

**FIGURE 3:**
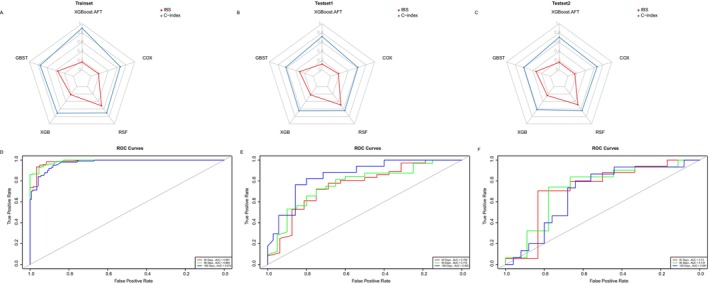
Performance comparison of different machine learning models. (A–C) Radar charts display the Concordance Index (C‐index) performance of models constructed using different algorithms for predicting patient mortality: (A) training cohort, (B) test cohort 1, (C) test cohort 2. (D–F) Time‐dependent Receiver Operating Characteristic (ROC) curves for different algorithms in the (D) training cohort, (E) test cohort 1, and (F) test cohort 2. AFT, Accelerated Failure Time; C‐index, Concordance Index; COX, Cox proportional hazards regression; GBST, Gradient Boosting Survival Tree; RSF, Random Survival Forest; XGB, eXtreme Gradient Boosting.

**FIGURE 4:**
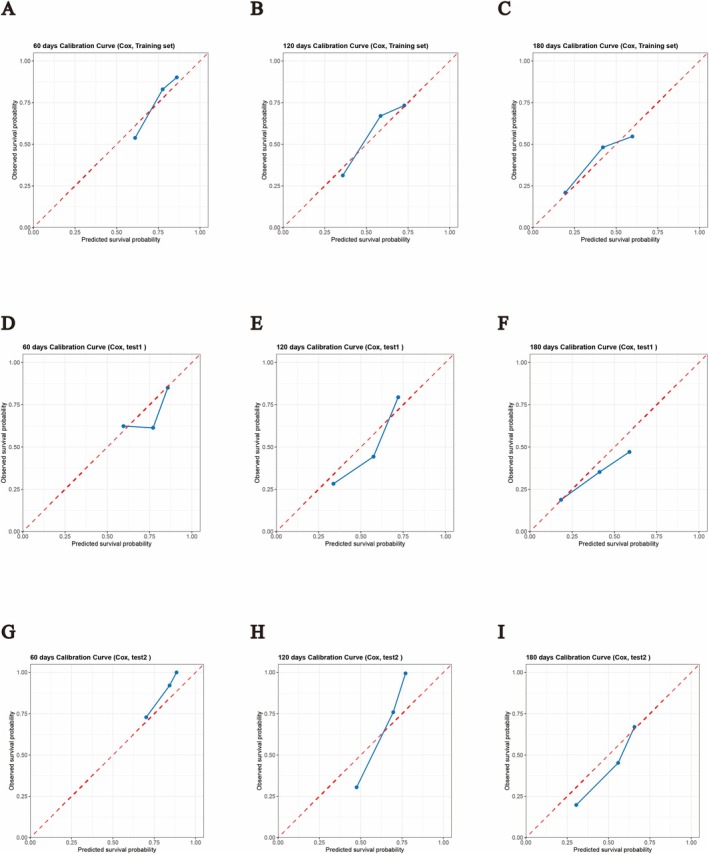
Calibration curves of the XGBoost AFT model at different time points. (A–C) Calibration curves for the training cohort at 60, 120, and 180 days. (D–F) Calibration curves for test cohort 1 at 60, 120, and 180 days. (G–I) Calibration curves for test cohort 2 at 60, 120, and 180 days. The diagonal line represents perfect prediction, and closer proximity of the curve to the diagonal indicates better calibration. The Brier Score (BS) for the corresponding cohort and time point is shown in each subplot title, with lower BS values indicating better predictive accuracy.

The models were constructed using the following five algorithms: GBST, XGBoost, XGBoost AFT, RSF, and CoxPH regression. The model performance was evaluated using the C‐index values for the training and test cohorts. The XGBoost AFT model exhibited the best performance, with C‐index values of 0.902, 0.722, and 0.705 for the training cohort, test cohort 1, and test cohort 2, respectively (Figure 3a–c). To quantify calibration performance during external validation, calibration curves were plotted and calibration slopes were calculated. Despite a moderate attenuation in the C‐index, the XGBoost AFT model maintained acceptable concordance between predicted and observed survival probabilities across both external validation cohorts (Figure 4). The ROC curves of the XGBoost AFT model in both the training and testing cohorts also demonstrated favorable discriminatory power (Figure 3d–f). To evaluate model performance more comprehensively, we calculated the Integrated Brier Score (IBS) for each model across different cohorts. The XGBoost AFT model achieved IBS values of 0.125 and 0.1616 in test cohort 1 and test cohort 2, respectively, which were substantially lower than those of other comparative models (e.g., GBST: 0.3129 and 0.3281; RSF: 0.4939 and 0.4868 in the respective cohorts), indicating its superior overall predictive accuracy and calibration (Figure 4). Compared with the conventional multivariable CoxPH model, the XGBoost AFT model achieved superior C‐indices in both the training set and the two external validation sets (where the CoxPH model yielded indices of 0.823, 0.686, and 0.679, respectively). This demonstrates the XGBoost AFT model's incremental advantage in capturing complex non‐linear relationships. Detailed comparative data are provided in the Results section (Figure 3, Supplementary Figure S3). The detailed parameter configuration of the optimal XGBoost AFT model is provided in Table S1. To assess the consistency of the model's performance across primary malignancies, we conducted a sensitivity analysis by stratifying patients into two subgroups: pancreatic cancer and non‐pancreatic biliary malignancies (including cholangiocarcinoma, gallbladder cancer, and ampullary cancer). The analysis revealed that the XGBoost AFT model sustained stable discriminative power within these subgroups (Figure S2).

### 3.3 Explainability

**FIGURE 5:**
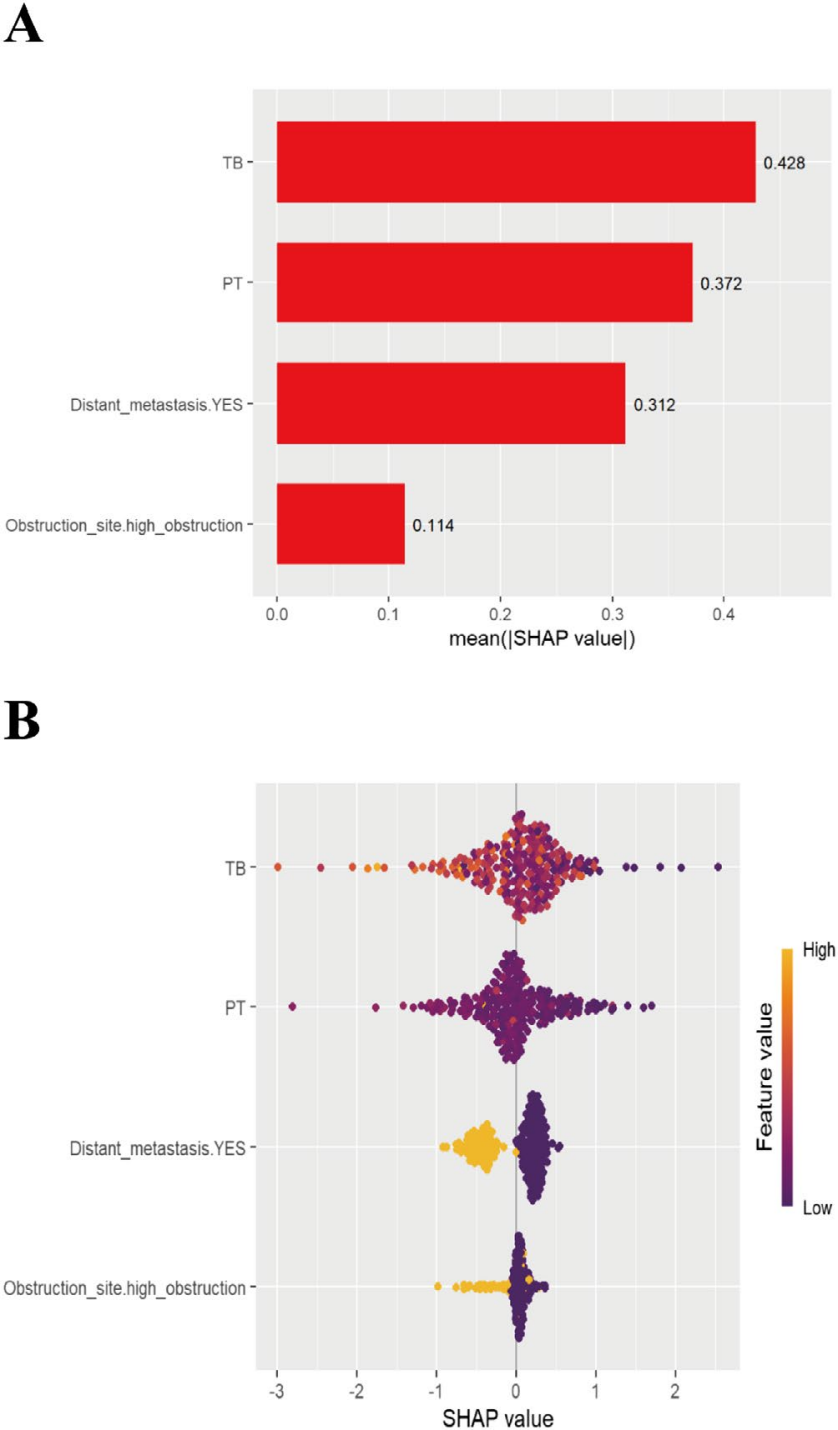
Visualization and explanation of machine learning models. (A) The Shapley additive explanations (SHAP) bar chart shows the weights of the four most important characteristics of the model. (B) The SHAP beeswarm plot shows how each feature impacts the predicted probability, with red and blue indicating an association with longer and shorter survival, respectively. PT, prothrombin time; TB, total bilirubin.

**FIGURE 6:**
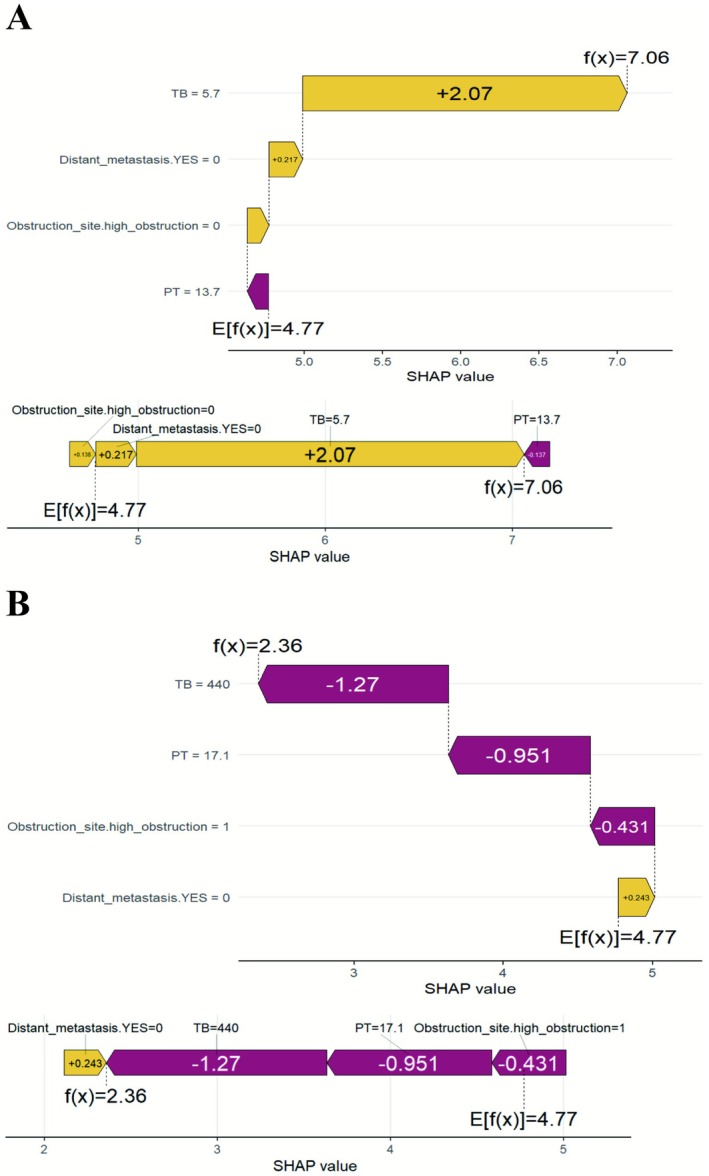
The SHapley Additive Explanation framework for individual predictions. (A) Projection of a case with a longer predicted survival time. (B) Projection of a case with a shorter predicted survival time. PT, prothrombin time; TB, total bilirubin.

To enhance the interpretability and clinical applicability of the XGBoost AFT model, we calculated the overall and individual SHAP values. An overview of the importance weights for the four key features of the model, tumor site, distant metastasis, total bilirubin, and prothrombin time, are shown in the SHAP bar chart (Figure 5a). The average SHAP values were 0.114 for tumor site, 0.312 for distant metastasis, 0.428 for total bilirubin, and 0.372 for prothrombin time; total bilirubin had the highest importance weight. The SHAP beeswarm plot (Figure 5b) illustrates the contribution of each feature to the predicted outcome. Specifically, low prothrombin time and tumor location in the lower part were associated with longer survival, whereas high total bilirubin levels and the presence of distant metastasis were associated with shorter survival. Four typical examples of correct predictions for patients at high risk and low risk are shown in Figure 6. The SHAP decision plot highlights how each feature contributes to the prediction for individual cases. The *E*[*f*(*x*)] value represents the model's baseline predicted probability and the *f*(*x*) value denotes the final predicted probability.

**FIGURE 7:**
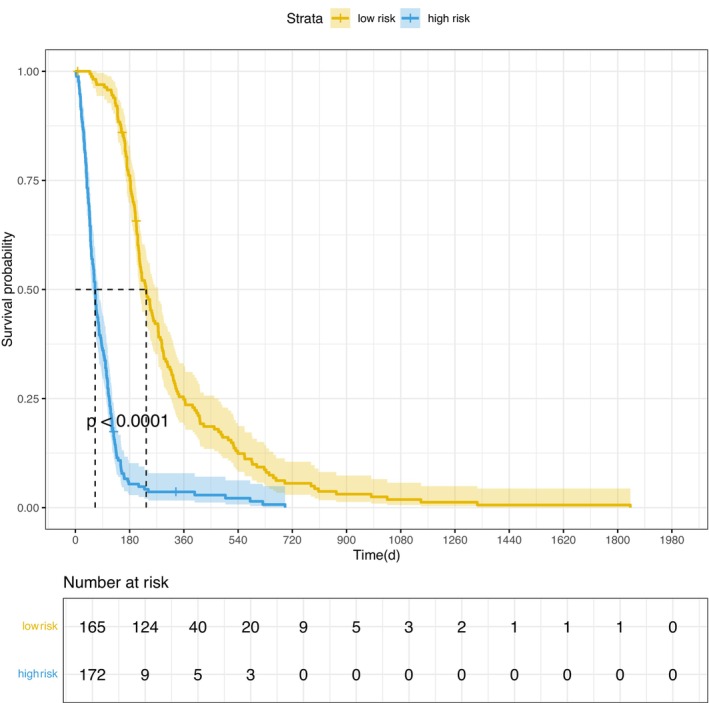
Kaplan–Meier curves of the different risk groups.

To evaluate the prognostic stratification value of the model [33], patients were categorized into the high‐risk (XGBoost AFT < 144.1461) and low‐risk (XGBoost AFT > 144.1461) groups based on the model's cutoff value, which was determined to maximize the Youden index. Based on this optimal threshold, the median survival time was 65 days for the high‐risk group compared to 232 days for the low‐risk group. A Kaplan–Meier survival analysis (Figure 7) confirmed that the model effectively stratified patients (*p* < 0.0001).

We developed an online tool to improve the user‐friendliness of the model. Users can easily obtain predicted risk scores by entering the specific characteristics of a patient. The model is available at https://zzd1076803421.shinyapps.io/modle11/.

## 4 Discussion

Prognostic outcomes can be optimized with the use of personalized treatment and by focusing on high‐risk patients. During this study, we investigated risk factors that influence postoperative survival in patients with MBO, developed a predictive model based on these factors, and validated the model's performance using external data. Of note, the XGBoost AFT model demonstrated excellent performance in the training cohort (C‐index: 0.902) and maintained acceptable discriminatory power in the external validation cohorts (Test cohort 1: 0.722; Test cohort 2: 0.705). This performance decay may be attributed to overfitting of specific patterns within the training data and inherent inter‐center heterogeneity. These findings highlight several study limitations: the retrospective design and the relatively modest sample size of the external validation cohorts (*n* = 92), all originating from the same geographic region. Such factors may constrain the model's generalizability; thus, further validation in larger, multi‐ethnic, and international populations is warranted. Nevertheless, the model retained a certain degree of generalizability in the external validation sets. Furthermore, the XGBoost AFT model achieved significantly lower Integrated Brier Scores in the test cohorts compared to other models, indicating superior overall predictive accuracy and calibration.

Our findings align with those in the existing literature. Previous studies have consistently demonstrated that distant metastasis is a critical factor that affects survival and that patients with metastatic tumors experience significantly short survival times [34]. In particular, liver metastasis has been identified as an independent factor that affects the 12‐month survival rate in patients with unresectable hilar cholangiocarcinoma [35]. Additionally, a prospective study involving 92 patients with obstructive jaundice due to hilar cholangiocarcinoma, treated with percutaneous transhepatic bile duct stenting, found that tumor staging is a crucial independent risk factor for survival; patients with advanced‐stage disease have notably shorter survival times than those of patients with early‐stage disease [36]. High‐level obstruction has also been recognized as an independent risk factor for poor jaundice reduction outcomes at 4 to 7 days and 1 month after surgery [37, 38]. High‐level obstruction, caused by tumor invasion of the common bile duct or a higher area, often results in multiple biliary obstructions and increases the surgical complexity and risk due to its proximity to the hepatic hilum. Furthermore, the complexity of the obstruction—such as unilateral versus bilateral involvement and the presence of concurrent gastric outlet obstruction (GOO)—may significantly influence prognosis, a factor frequently under‐represented in MBO clinical studies [39]. High total bilirubin levels before stenting can significantly affect patient survival; levels > 14 mg/dL have been associated with higher 30‐day mortality rates [40]. High preoperative bilirubin levels indicate severe and prolonged biliary obstruction that can compromise the effectiveness of bile drainage after stenting [41]. Contrary to some studies suggesting that albumin levels correlate with patient survival [42], our study found that albumin level did not significantly affect survival. This discrepancy may be attributable to the sensitivity of albumin to external factors; therefore, albumin could be less reliable than other indicators when used to evaluate the perioperative nutritional status and liver function. Through a large sample analysis and a comprehensive multivariable model, this study reinforced the significance of factors such as bilirubin level, prothrombin time, obstruction level, and distant metastasis in predicting survival outcomes for patients with MBO. Our findings indicate that high bilirubin level, prolonged prothrombin time, high obstruction, non‐ampullary tumor types, and the presence of distant metastasis are associated with a poor prognosis, thus underscoring the need for increased vigilance regarding the patients' conditions.

The predictors identified through SHAP analysis have direct clinical significance. For patients presenting with profound hyperbilirubinemia, proactive biliary decompression (e.g., percutaneous transhepatic biliary drainage) or hepatoprotective therapy may be prioritized prior to ERCP to improve survival outcomes. In cases of prolonged prothrombin time, pre‐procedural correction via vitamin K supplementation or plasma transfusion is essential. Patients with high‐level (hilar) obstructions require meticulous preoperative imaging to optimize drainage strategies. Crucially, this model provides a framework for multidisciplinary team (MDT) decision‐making: high‐risk patients may benefit from integrating systemic therapies (chemotherapy or targeted agents) with biliary drainage, whereas low‐risk patients could be managed more aggressively with primary drainage alone. Such a risk—stratification strategy is expected to optimize resource allocation and enhance prognosis. We acknowledge that pooling diverse malignancies (pancreatic cancer, cholangiocarcinoma, gallbladder cancer, and ampullary cancer) may introduce biological heterogeneity. However, our rationale for a unified model is rooted in the clinical reality that for patients with MBO, palliative ERCP decision‐making and early postoperative prognosis are primarily driven by obstruction‐related physiological derangements (e.g., jaundice and coagulopathy) rather than histological subtype alone. Sensitivity analyses confirmed consistent model performance across pancreatic and non‐pancreatic subgroups (Figure S2), supporting its utility in predicting obstruction‐related mortality. Nevertheless, given the limited sample sizes for specific subtypes like ampullary cancer, these findings should be considered exploratory. Developing histology‐specific models in larger cohorts remains a future objective to refine predictive precision.

Compared to traditional Cox proportional hazards models, ML models offer greater flexibility and do not rely on assumptions of data distribution [43]. Although ML models are often criticized as “black boxes,” advancements in interpretability techniques, such as the SHAP method, have contributed to more accessible and understandable results [25, 31, 44]. The SHAP method helps visualize the overall and individual contributions of each feature, thereby facilitating clinical application and enhancing clinicians' confidence in using predictive models [25, 32, 45, 46]. In our model, SHAP values were used to represent the weights and effects of four key predictive features, illustrating their contributions to the final SHAP values. By identifying these key predictors, clinicians can conduct more accurate risk assessments before surgery, thereby aiding in the understanding of potential risks and benefits and informing decisions regarding personalized treatment plans, such as whether to proceed with surgery or consider adjuvant therapy.

Nevertheless, this study has some limitations. Being a retrospective analysis, it may have been subjected to selection bias. The use of interpolated values through multiple imputation introduces potential estimation errors that could have affected the ML model's performance. The XGBoost AFT model demonstrated excellent performance in the training set (C—index of 0.902), but there was a decline in external validation (0.722 and 0.705), suggesting overfitting and inter—center heterogeneity. Differences in patient characteristics, diagnosis and treatment procedures between the training and validation cohorts may limit the generalization of the model. Furthermore, discrepancies in follow‐up periods and the distribution of survival times between the training and test sets may lead to fluctuations in predictive accuracy across different time points and affect the assessment of long‐term prediction performance. Additionally, the relatively limited sample size of the external test cohorts, particularly the small number of events at longer‐term follow‐up points, constrains the accurate evaluation of the model's long‐term predictive performance. In addition, as all data were from a single country, further multi—ethnic and multi—center validation is needed to improve universality. Systemic differences in equipment, procedures, and population characteristics among the three participating centers may also introduce bias. Although we tried to control this through data standardization and external validation, center heterogeneity remains a potential limitation. Patients with concurrent gastric outlet obstruction (GOO) or prior reconstructive gastrointestinal surgery (e.g., Roux‐en‐Y anatomy) were excluded from this study. These populations often necessitate advanced techniques, such as endoscopic ultrasound‐guided biliary drainage (EUS‐BD) [47, 48], and exhibit distinct pathophysiology. Given that MBO is heterogeneous regarding obstruction morphology (e.g., hilar vs. distal, or presence of GOO), which may confound prognostic assessments [39], our model currently applies primarily to simple MBO. Its validity in complex or mixed obstruction patterns requires further investigation. Immunonutritional indicators (such as lymphocyte count and prognostic nutritional index) were not included due to inconsistent recording standards among centers, yet they are closely related to tumor prognosis, and their absence may limit the model's performance. Critically, due to incomplete and heterogeneous documentation across participating centers, we were unable to incorporate data on adjuvant therapies (chemotherapy, targeted therapy, or radiotherapy). The omission of these treatments, which substantially modulate survival, constitutes a significant limitation that may impact the model's predictive accuracy and clinical utility. Consequently, future prospective studies are essential to systematically record and integrate therapeutic regimens into the predictive framework. The combined modeling of multiple tumor types (pancreatic cancer, cholangiocarcinoma, gallbladder cancer, and ampullary cancer) was based on their shared clinical background of biliary obstruction and ERCP intervention [49, 50]. Although the primary tumor type did not reach a significant threshold in variable screening (possibly due to sample size limitations), biological differences may lead to the masking of unique prognostic factors. In the future, a larger sample size is required to conduct stratification analysis by pathological type or develop subtype—specific prediction tools. Despite advancements in the SHAP method for model interpretability, ML models, particularly complex ones, are still associated with challenges related to understanding and explaining decision‐making processes. Additionally, this study did not address some potentially influential factors such as genetic mutations and the immune status, which may affect the prognosis of MBO. Future research should focus on integrating molecular biomarkers, radiomic features, and advanced imaging metrics into machine‐learning frameworks to enhance prognostic precision and facilitate personalized precision medicine. Additionally, multicenter, large‐scale prospective studies should be conducted to enhance the reliability and generalizability of the results. Interpretability techniques should be enhanced to provide clearer insights regarding the decision‐making processes of complex ML models, thus aiding in their clinical adoption and application. Recent advances in molecular biology and immunology should be integrated in further studies to explore the benefits of combined treatment strategies for patients with MBO.

## 5 Conclusions

This study systematically analyzed the risk factors affecting postoperative survival in patients with MBO, developed a predictive model, and validated the superior performance of that model over other models using external datasets. Despite some limitations, the findings offer valuable insights regarding the postoperative management and personalized treatment of patients with MBO. Future research is essential for improving the prognosis and enhancing the quality of life in individuals with MBO.

## Author Contributions

All authors contributed to the study conception and design. Material preparation and data collection and analysis were performed by ZDZ, CYB, LJZ, WWL, and KXH. The first draft of the manuscript was written by ZDZ. HBL and QGS commented on subsequent versions of the manuscript. All authors read and approved the final manuscript.

## Funding

The work was funded by grants from the Science and Technology Project of Zhejiang Medical and Health [2025KY1589], Key Discipline of Hepatobiliary and Pancreatic Surgery of Jiaxing City [2023‐zc‐005], Translational Therapy Center for Hepatobiliary Pancreatic Cancer [2021‐YJZX‐04], Science and Technology Project of Jiaxing [2022 AD30066, 2023 AD31059], Jointly Cultivate Disciplines of Jiaxing City and Zhejiang province [2023‐PYXK‐001], and National Clinical Key Specialty Construction Project [2023‐GJZK‐001].

## Ethics Statement

This study protocol was approved by the institutional review boards of The First Hospital of Jiaxing (no. 2022‐LY‐229), The Second Hospital of Jiaxing (no. 2023‐ZFYJ‐066), and the Affiliated Hospital of Shaanxi University of Traditional Chinese Medicine (no. 2023‐SZFYIEC‐PJ‐21). All procedures followed the ethical standards of the responsible committee on human experimentation (institutional and national) and the Helsinki Declaration of 1964 and later versions.

## Consent

A waiver of the informed consent was approved by the institutional review boards of The First Hospital of Jiaxing, The Second Hospital of Jiaxing, and the Affiliated Hospital of Shaanxi University of Traditional Chinese Medicine because of the retrospective nature of the study.

## Conflicts of Interest

The authors declare no conflicts of interest.

## Supporting information


**Figure S1:** Distribution of key continuous predictors and identification of extreme values.

**Figure S2:** Comparison of model discriminative performance (C—index) among different cohorts and subgroups before and after sensitivity analysis.

**Figure S3:** Calibration curve of the multivariate Cox proportional—hazards model.

**Table S1:** Final parameter configuration of the optimal XGBoost AFT model.

## Data Availability

The data used and/or analyzed during the current study are available from the corresponding author upon reasonable request.
