# Supplementary figures and images for: Predicting Postoperative Survival in Patients With Malignant Biliary Obstruction Using an Interpretable Machine Learning Model: A Multicenter Study
Source: Cancer Med. 2026 Mar 6;15(3):e71692. doi: 10.1002/cam4.71692 (PMC12964315; doi:10.1002/cam4.71692)

Boxplot of Original Values

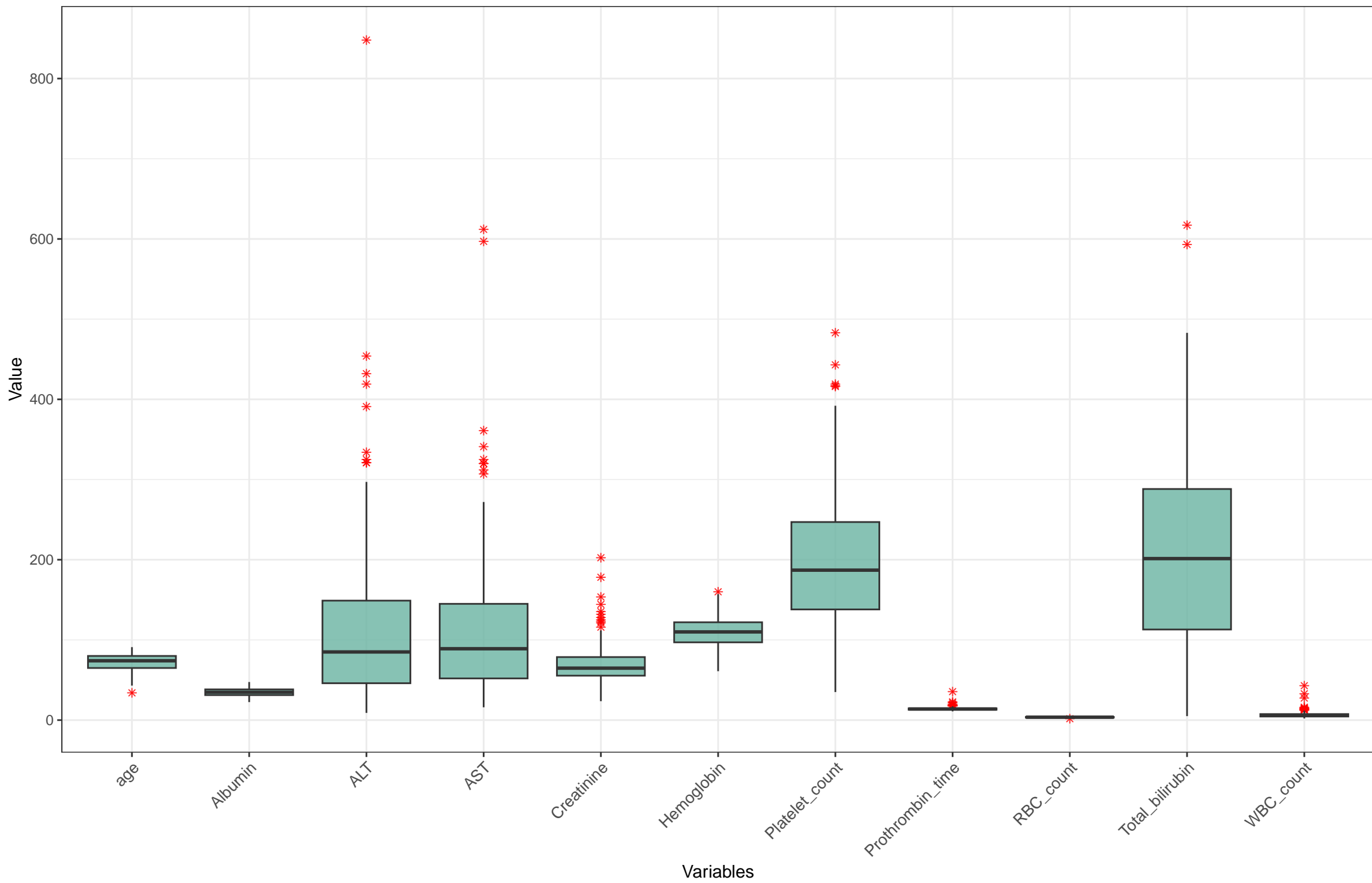

Boxplot after Standardization (Z-score)

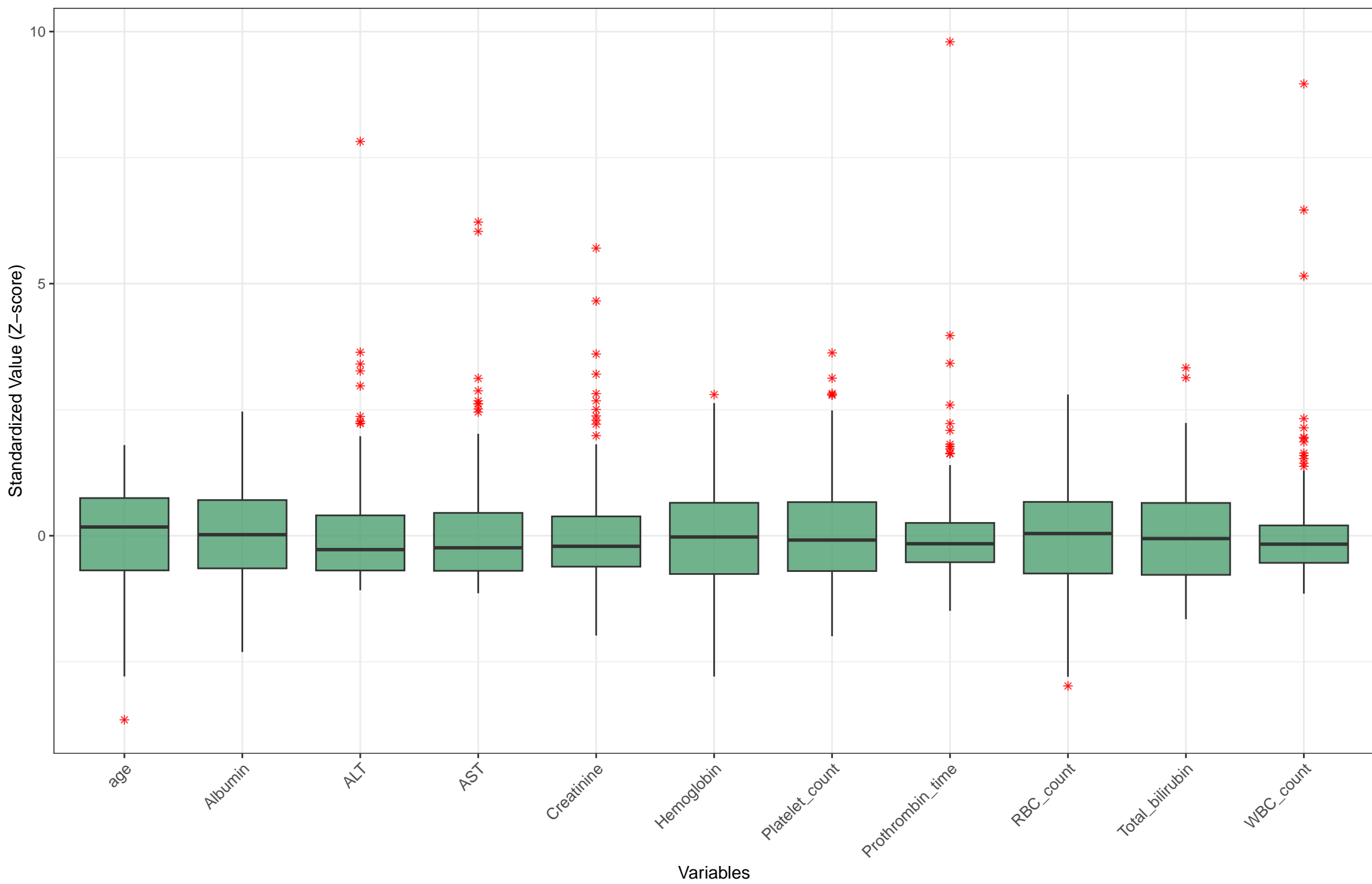

Supplement: Supplementary file 1 — Figure S1: Distribution of key continuous predictors and identification of extreme values. [file CAM4-15-e71692-s003.pdf]

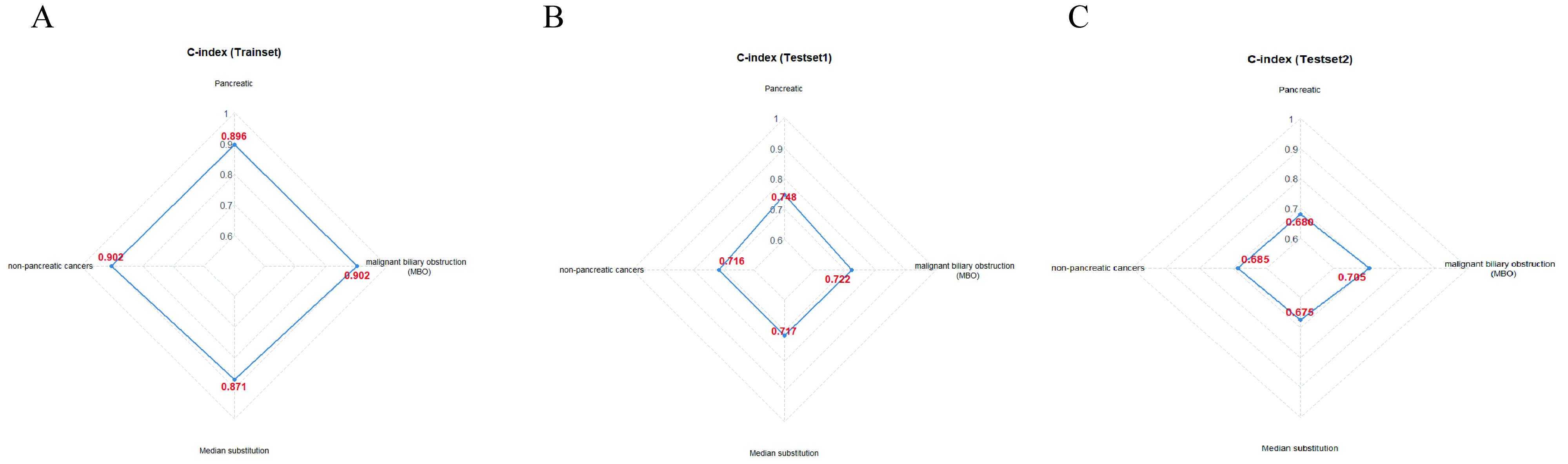

Supplement: Supplementary file 2 — Figure S2: Comparison of model discriminative performance (C—index) among different cohorts and subgroups before and after sensitivity analysis. [file CAM4-15-e71692-s002.tif]

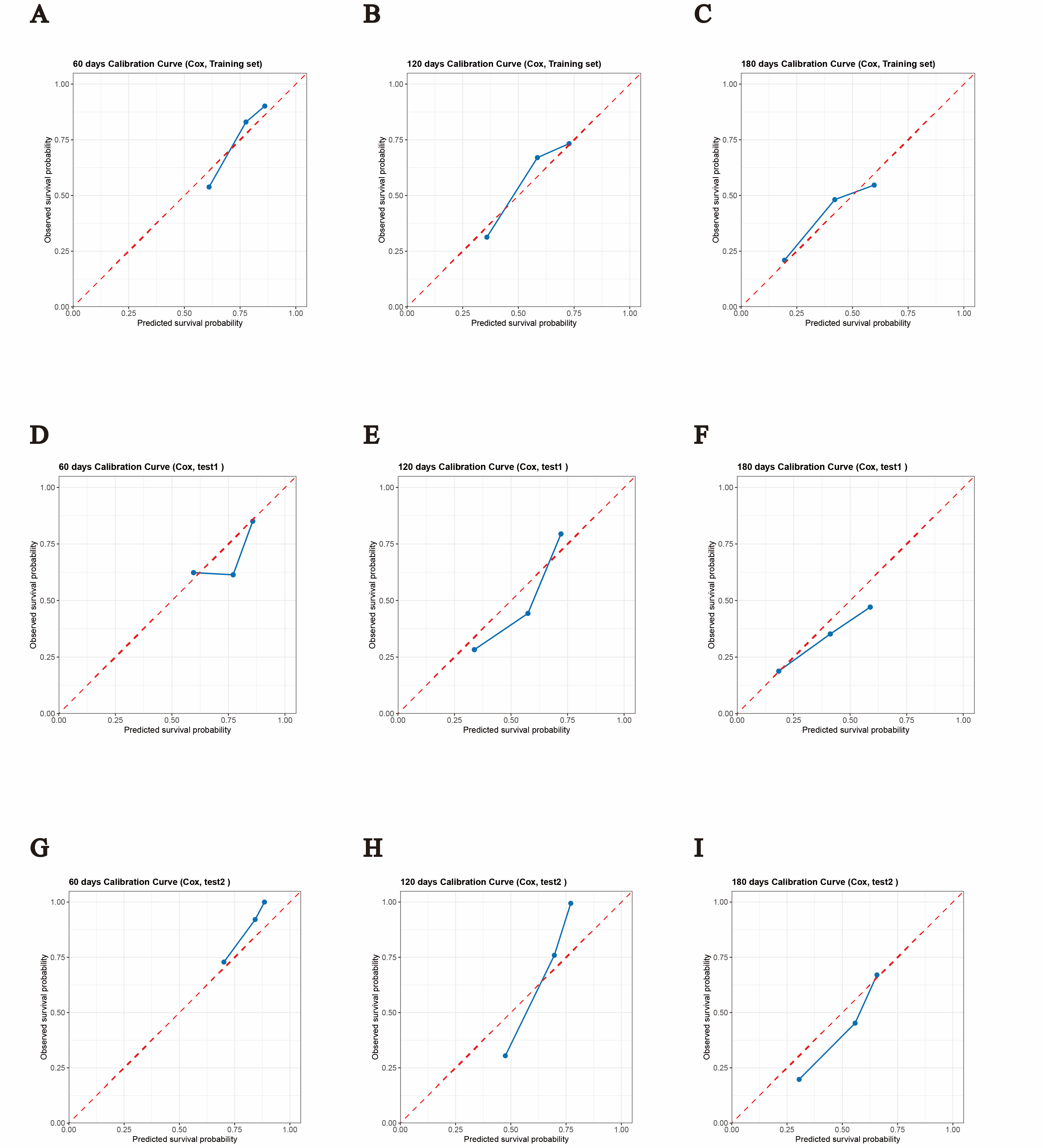

Supplement: Supplementary file 3 — Figure S3: Calibration curve of the multivariate Cox proportional—hazards model. [file CAM4-15-e71692-s004.tif]
