# Supplementary material for: Predicting Postoperative Survival in Patients With Malignant Biliary Obstruction Using an Interpretable Machine Learning Model: A Multicenter Study
Source: Cancer Med. 2026 Mar 6;15(3):e71692. doi: 10.1002/cam4.71692 (PMC12964315; doi:10.1002/cam4.71692)
Supplement: Supplementary file 4 — Table S1: Final parameter configuration of the optimal XGBoost AFT model. [file CAM4-15-e71692-s001.docx]

| Hyperparameter | Value |
| --- | --- |
| objective | survival:aft |
| eval_metric | aft-nloglik |
| aft_loss_distribution | normal |
| aft_loss_distribution_scale | 0.1 |
| tree_method | hist |
| learning_rate | 0.5 |
| max_depth | 5 |
| nrounds | 1000 |
| early_stopping_rounds | 10 |
| min_child_weight | 1 |
| gamma | 0 |
| subsample | 1 |
| colsample_bytree | 1 |
| lambda | 1 |
| alpha | 0 |
| max_delta_step | 0 |
| grow_policy | depthwise |
| scale_pos_weight | 1 |
| verbosity | 1 |
| booster | gbtree |
